# Nonlinear restoration of pulse and high noisy images via stochastic resonance

**DOI:** 10.1038/srep16183

**Published:** 2015-11-04

**Authors:** Qibing Sun, Hongjun Liu, Nan Huang, Zhaolu Wang, Jing Han, Shaopeng Li

**Affiliations:** 1 State Key Laboratory of Transient Optics and Photonics, Xi’an Institute of Optics and Precision Mechanics of CAS, Xi’an, 710119, China

## Abstract

We propose a novel scheme for restoring pulse and high noisy images using stochastic resonance, which is based on the modulation instability and provides a cross-correlation gain higher than 8. As opposed to previously reported designs, this unique approach employs a continuous noise and pulse signal for the generation of modulation instability. The visibility and quality of output images can be improved by appropriately adjusting the system parameters. This provides a simple and feasible method for detecting low-level or hidden pulse images in various imaging applications.

In the fields of optical signal detection and processing, the low-level signal is usually overwhelmed by noise and difficult to be distinguished. Noise is usually considered harmful and degrades the performance of dynamical systems. However, in some special nonlinear systems, the presence of noise enhances the detection ability of weak signals, displaying the phenomenon of stochastic resonance^1^. This makes it possible to detect the noise-hidden signals, which is impossible in conventional linear systems. It is also appropriate for the signals hidden by the noise with approximate frequencies. With the development of stochastic resonance, it now appears in a wide variety of physical systems, which is suitable for restoring and self-filtering the weak signals^2^,^3^. The classic system that undergoes stochastic resonance is induced by a particle in a double-well potential. It is restricted to a threshold and usually focuses on one-dimensional beams rather than two-dimensional images^1^, while there is no need for feedback or threshold in the stochastic resonance based on modulation instability^4^,^5^,^6^. As with other kinds of stochastic resonance, signal amplification occurs at the expense of the noise background, leading to reconstruction of the noise-hidden images^3^. Nowadays, the nanosecond pulse images have been widely used in optical processing and are usually low-level in remote light detection and transmission^7^,^8^,^9^,^10^. Considering the rapid development of the pulse images and their practical applications, it is urgent to investigate the stochastic resonance with a simple structure and flexible design, which will promote the detection sensitivity of weak and high noisy images.

In this paper, we report a practical scheme for restoring pulse and high noisy images via stochastic resonance, which exhibits a high cross-correlation gain for certain parameter ranges. This dynamical stochastic resonance based on modulation instability is generated and hold with coupling between the continuous incoherent noise and coherent pulse signal. The nanosecond noise-hidden images grow at the expense of noise and become visible by optimizing the system parameters. This suggests a valuable design for restoring pulse images through stochastic resonance in high noisy environments.

## Results

### Experimental setup and design

**Figure 1:**
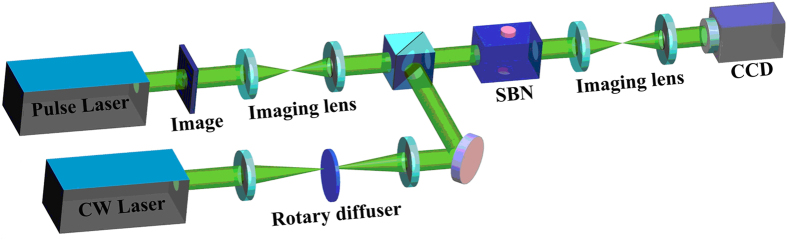
Scheme for restoring pulse and high noisy images via stochastic resonance.

In view of the practical applications in laser radar and other imaging fields, a more flexible and versatile design is used to restore and reconstruct the pulse noise-hidden images. It consists of three key elementary components: a nanosecond pulse signal carrying an image, a continuous incoherent noise and a nonlinear system containing a photorefractive crystal. Figure 1 presents details of the scheme employed in our work, in which the stochastic resonance is generated by modulation instability. A distinguishing feature is that there is no need for time synchronization between the pulse signal and continuous noises, which is vital in applications. The pulse signal is a purely coherent nanosecond image of a number “20” formed by a 4-f optical system and Q-switched Nd:YAG laser. Its wavelength, repetition frequency and pulse width are 532 nm, 1 kHz and about 15 ns, respectively. The continuous noise is a spatially incoherent light generated from a “Lens-Diffuser-Lens” system, which is irradiated by a continuous laser frequency doubled Nd:YAG laser. A 5.5×5×10 mm^3^ SBN:75 photorefractive crystal doped with CeO_2_ is used as the nonlinear medium, whose nonlinear coefficient is controlled by tuning the applied voltage parallel to its optical axis. The pulse signal and continuous noise are simultaneously and collinearly injected into the SBN:75 photorefractive crystal. To verify the extraction ability of the stochastic resonance under the high noisy background, the initial signal-to-noise intensity ratio is fixed at about 1:25 with the signal power of about 150 nW. Coupling between the signal and noise takes place during the stochastic resonance process and light exiting the photorefractive crystal is then imaged onto a CCD camera.

### Pulse output images in theory

Considering the dynamics of the low-level signals, we take the instability-linear perturbation theory and focus on the response of the noise to the driving signal which is more appropriate for the incoherent dynamics^10^,^11^,^12^. The perturbation is linearized to treat each pixel individually for the low-level intensity of input pulse images^10^. Neglecting the linear loss and the high-order nonlinear contribution, the growth rate g can be described as^3^,^10^


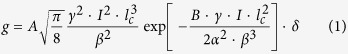


where *A* and *B* are effective mode-dependent normalization constants giving the height and location of the visibility peak, *γ* is the nonlinear coefficient of spatial coupling electric field, *β* is the diffraction coefficient, α is the wavenumber, *l*_*c*_ is the correlation length, *I* is the incident light intensity, *δ* indicates the temporal distribution of nanosecond pulse, respectively.

**Figure 2:**
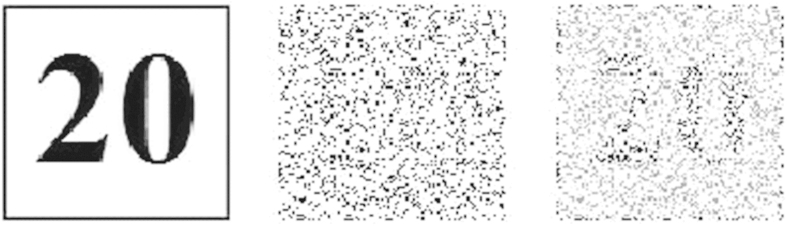
(**a**) Original image, (**b**) pulse and high noisy image (**c**) output image.

**Figure 3:**
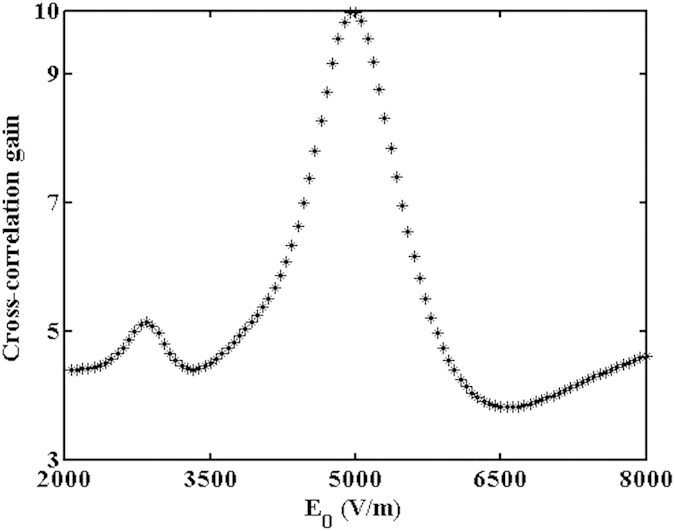
Cross-correlation gain varies with the applied voltage.

According to equation (1), the nanosecond output image via stochastic resonance is numerically calculated and analyzed. The input signal-to-noise intensity ratio is fixed at about 1:25 and namely the image is highly noise-hidden. The output image versus the nonlinearity for a fixed correlation length is presented in Fig. 2. As the applied voltage increases, the output signal-to-noise intensity ratio increases until a maximum is reached, which makes the output image becomes more visible and distinct. This is caused by the instability of energy coupling between the weak signals and random noises. The distribution of total energy of the coherent signal and incoherent noise is reconstructed due to the movement of particles as the nonlinear index change Δn keeps acting on the interface. At last, the extracted image becomes most noticeable at an optimal voltage, as shown in Fig. 2(c). To describe the energy transfer of the nonlinear dynamical process, the normalized cross-correlation coefficient is used as a quantitative measure of the similarity between the input and output images^13^. As shown in Fig. 3, there exists an optimal voltage for acquiring the maximum cross-correlation gain through the stochastic resonance process. That is, the energy transferring efficiency from high-level noise to low-level signal reaches a maximum at an optimal applied voltage. However, for a higher Δn, the turbulent dynamics continues mixing signal and noise, the border of signal and noise will degrade in coherent-incoherent snake-like instability^14^. This suggests that the applied voltage and noise intensity are two important parameters of stochastic resonance, which influence the visibility and quality of the output image.

### Pulse output images in experiment

**Figure 4:**
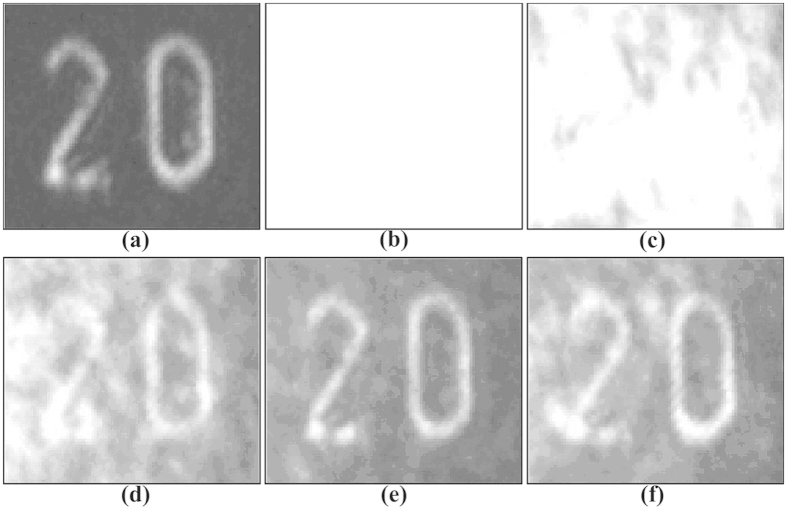
Output images at different applied voltages. (**a**) Pure image, (**b**) high noisy image, (**c**) 1000 V, (**d**) 1500 V, (**e**) 2000 V, (**f**) 2400 V.

**Figure 5:**
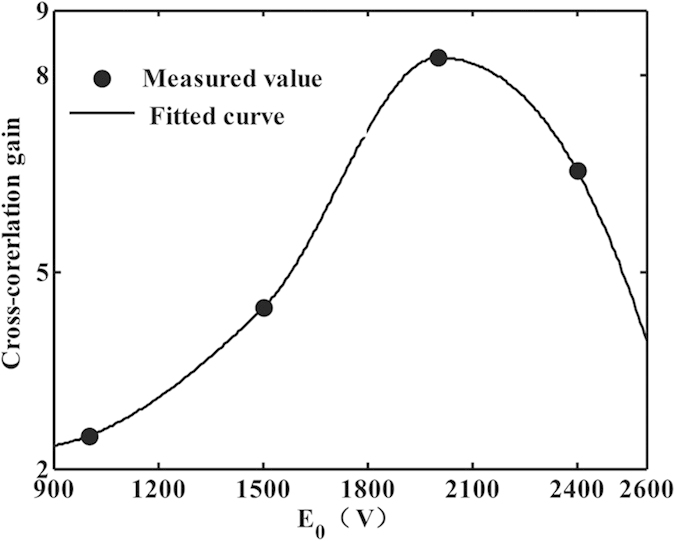
Relationship between cross-correlation gain and applied voltage.

The experiment for the pulse and high noisy image restoration via stochastic resonance was performed using the scheme shown in Fig. 1. The applied voltage across the crystal is used to adjust the nonlinear index change Δn of the photorefractive crystal^10^. The spatial correlation length of the continuous noise is determined by the spot size on the diffuser and its rotating speed. The visibility and quality of the output images are optimized by carefully tuning the applied voltage, as illustrated in Fig. 4. The pulse and high noisy images become more and more clear as the applied voltage increases to about 2000 V, at which the optimal reconstruction result appears. There is a positive exchange during the stochastic resonance process generated by modulation instability. The reason is that the nonlinear coefficient of the photorefractive crystal varies with the applied voltage, and thus the fractional nonlinear index change Δn can be adjusted. The weak pulse signal and large continuous noise together means that the initial signal is essentially a seed that triggers an instability in the noise. Specially, the very weak signal first seeds a potential that concentrates the noise, in turn, nonlinear coupling amplifies the signal and reinforces the potential. This leads to the signal reconstruction through seeded instability, where intensity perturbations grow at the expense of a uniform background. Namely, nonlinearity can improve the visibility of the signal, while too much nonlinearity will degrade the resonance pattern and destroy the visibility. As shown in Fig. 4(f), the output image becomes blurred again when the applied voltage is increased further. Meanwhile, the cross-correlation gain is also analyzed as depicted in Fig. 5. An optimal cross-correlation gain higher than 8 is obtained at the applied voltage of 2000 V, whose variation trend is similar to the theoretical results.

**Figure 6:**
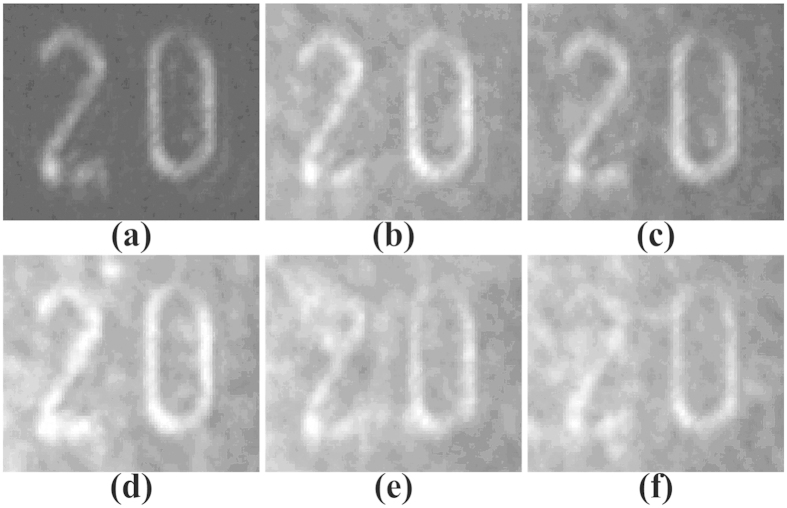
Output images at different signal-to-noise intensity ratios at a fixed applied voltage of 2000 V. (**a**) Pure image, (**b**) 1:15, (**c**) 1:20, (**d**)1:25, (**e**) 1:30, (**f**) 1:35.

The initial signal-to-noise intensity ratio is another important parameter that determines the performance of the stochastic resonance. Figure 6 details its impact on the output image quality at a fixed applied voltage of 2000 V. It is clearly seen that the output pulse image is most visible at the initial intensity ratio of about 1:25 and becomes blurred as the initial intensity ratio increases further. That is, the modulations become more pronounced with higher visibility at an appropriate signal-to-noise intensity ratio. However, as the initial intensity ratio continues to increase, too much noise will dominate this system and destroy the conditions of stochastic resonance, which leads to the distortion of output images. This typical signature is caused by the instability of energy coupling between weak signals and random noise^1^,^3^,^10^. In a word, all the above results strongly indicate that the applied voltage and noise intensity are two key parameters influencing the properties of the stochastic resonance.

**Figure 7:**
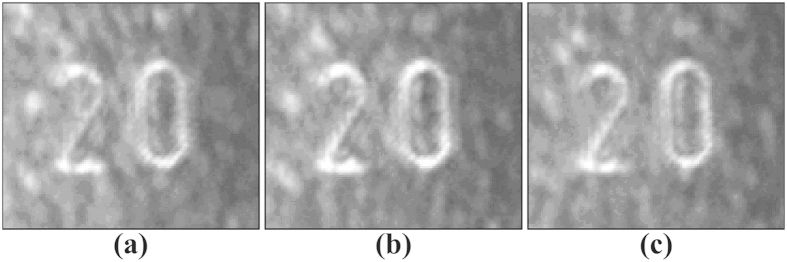
Output images at different repetition frequencies. (**a**) 100 Hz, (**b**) 500 Hz, and (**c**) 1 kHz.

According to the analysis above, at a fixed voltage of 2000 V and signal-to-noise intensity ratio of 1:25, the influence of the repetition frequency on the property of the output image is analyzed as detailed in Fig. 7. The output images are recorded at the repetition frequencies of 100 Hz, 500 Hz and 1 kHz, respectively. The signal images are effectively restored from the high-level noise, in which the cross-correlation gains are all higher than 8 at the three repetition frequencies. This indicates that the reconstruction of pulse noise-hidden images via stochastic resonance is applicable at a large repetition frequency range. This characteristic broadens the application fields of the stochastic resonance.

**Figure 8:**
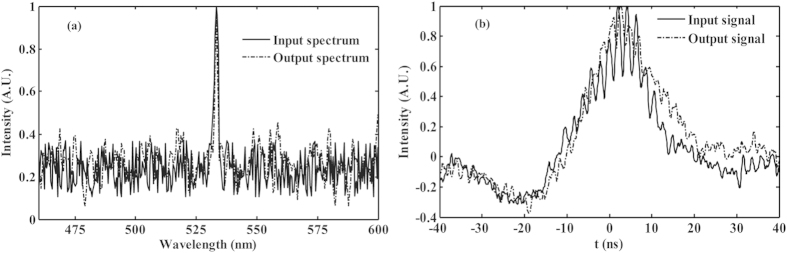
Output (a) spectrum and (b) waveform via the stochastic resonance.

In addition, the output spectrum and temporal profile are monitored by a HR2000 spectrometer and a Tektronix oscilloscope, respectively. As evident from Fig. 8, the output spectrum and temporal profile are almost the same as that of the input signal. That is to say, there is no distortion during the stochastic resonance process, which is important in the applications. The minor difference between them is mainly caused by the output stability of the nanosecond pulse laser. The power fluctuations of the pulse envelope are induced by the longitudinal mode beating^15^. These characteristics are able to meet the requirements for detecting low-level and pulse noisy images in many research fields.

## Discussion

The stochastic resonance produced by modulation instability is held with coherent-incoherent coupling. This nonlinear coupling is necessary to cause the energy transfer between different components^3^. Diffraction and nonlinearity are the two necessary conditions to generate modulation instability in a nonlinear system^16^, which determine the intensity of incident wave enhanced or diminished in propagation. Tuning parameters suitably can contribute the energy transferring from the high-level noise to the low-level signal. The nanosecond noise-hidden images grow at the expense of noise and the output cross-correlation gain reaches a peak at appropriate system parameters, which reflects the phenomena of stochastic resonance. This means that stochastic resonance has very important application value in weak signal detection and processing.

In conclusion, we have demonstrated a flexible and compatible scheme for reconstructing the pulse and high noisy images via stochastic resonance. It does not need the time synchronization and allows of a large repetition frequency range, which extends the applications of stochastic resonance. The stochastic resonance can extract nanosecond images from the high noisy background with a cross-correlation gain higher than 8 by carefully adjusting the system parameters. This provides a potential method for recovering the original pulse images from noise-hidden images in various applications of pulse imaging process.

## Additional Information

**How to cite this article**: Sun, Q. *et al.* Nonlinear restoration of pulse and high noisy images via stochastic resonance. *Sci. Rep.* **5**, 16183; doi: 10.1038/srep16183 (2015).
